# Exome sequencing identifies a novel mutation of the *GDI1* gene in a Chinese non-syndromic X-linked intellectual disability family

**DOI:** 10.1590/1678-4685-GMB-2016-0249

**Published:** 2017-08-31

**Authors:** Yongheng Duan, Sheng Lin, Lichun Xie, Kaifeng Zheng, Shiguo Chen, Hui Song, Xuchun Zeng, Xueying Gu, Heyun Wang, Linghua Zhang, Hao Shao, Wenxu Hong, Lijie Zhang, Shan Duan

**Affiliations:** 1 Laboratory of Medical Genetics, Center for Birth Defect Research and Prevention, Shenzhen Research Institute of Population and Family Planning, Shenzhen City, People's Republic of China; 2 College of Pharmacy, Nankai University, Tianjin City, People's Republic of China

**Keywords:** Intellectual disability, *GDI1* gene, guanosine diphosphate dissociation inhibitor, whole exome sequencing

## Abstract

X-linked intellectual disability (XLID) has been associated with various genes. Diagnosis of XLID, especially for non-syndromic ones (NS-XLID), is often hampered by the heterogeneity of this disease. Here we report the case of a Chinese family in which three males suffer from intellectual disability (ID). The three patients shared the same phenotype: no typical clinical manifestation other than IQ score ≤ 70. For a genetic diagnosis for this family we carried out whole exome sequencing on the proband, and validated 16 variants of interest in the genomic DNA of all the family members. A missense mutation (c.710G > T), which mapped to exon 6 of the Rab GDP-Dissociation Inhibitor 1 (*GDI1*) gene, was found segregating with the ID phenotype, and this mutation changes the 237th position in the guanosine diphosphate dissociation inhibitor (GDI) protein from glycine to valine (p. Gly237Val). Through molecular dynamics simulations we found that this substitution results in a conformational change of GDI, possibly affecting the Rab-binding capacity of this protein. In conclusion, our study identified a novel *GDI1* mutation that is possibly NS-XLID causative, and showed that whole exome sequencing provides advantages for detecting novel ID-associated variants and can greatly facilitate the genetic diagnosis of the disease.

## Introduction

Intellectual disability (ID) is a neurodevelopmental disorder that appears before the age of 18. Its main clinical manifestations are intellectual deficits and social adjustment problems (Raymond, 2006). The average prevalence of ID in the last two decades was 13.0 per 1,000 in eight-year-old children in Atlanta, USA (Van Naarden Braun *et al.*, 2015), and 7.5 per 1,000 in the general population in China during the same period (Wu *et al.*, 2010). Impaired cognitive ability and social adaptation difficulties make it difficult for ID patients to live independently. Thus, the patients usually need lifelong care at home or in welfare centers, which pose enormous socioeconomic burdens for their family and the society (van Schrojenstein Lantman-de Valk and Walsh, 2008).

ID may arise from environmental factors, genetic predisposition, or a combination of both. In addition, the clinical manifestations of ID are highly heterogenic, which makes it difficult to confirm the etiology of most ID patients by traditional clinical diagnostic processes (Stevenson *et al.*, 2003). As to the cases in which genetic factors play a role, a great variety of chromosomal abnormalities and gene mutations are involved (Inlow and Restifo, 2004). Hitherto, there are approximately 820 genes considered responsible for ID (Kochinke *et al.*, 2016). Examining all of those genes in each ID case by Sanger sequencing is impractical. Therefore, there are increasing needs for new technical improvements to make precise molecular diagnosis for inherited ID patients. Nowadays, the next-generation sequencing (NGS) technology provides advantages for the genetic diagnosis of ID. The application of exome sequencing, a variant of NGS that focuses on the coding regions of the genome, improves the efficiency of molecular diagnosis and helps to uncover novel mutations present in either sporadic or familial cases with non-specific phenotypes (Topper *et al.*, 2011; de Ligt *et al.*, 2012; Rauch *et al.*, 2012).

Here, we present the genetic diagnosis of a Chinese family of Han origin with three males suffering from non-syndromic X-linked intellectual disability (NS-XLID) carried out by whole exome sequencing (WES). To our knowledge, this family might be the fifth XLID case caused by mutations located in the Rab GDP-Dissociation Inhibitor 1 (*GDI1*) gene (OMIM*300104) ever reported (Strobl-Wildemann *et al.*, 2011).

## Materials and Methods

### Ethical statement

This study was approved by the Ethics Committee of Shenzhen Research Institute of Population and Family Planning (SZIPP) in accordance with the Declaration of Helsinki (review list No. 20150411001). Written informed consents for publication of clinical information and genetic investigation were obtained from each participant or their legal guardians.

### Clinical information of patients and family ascertainment

**Figure 1:**
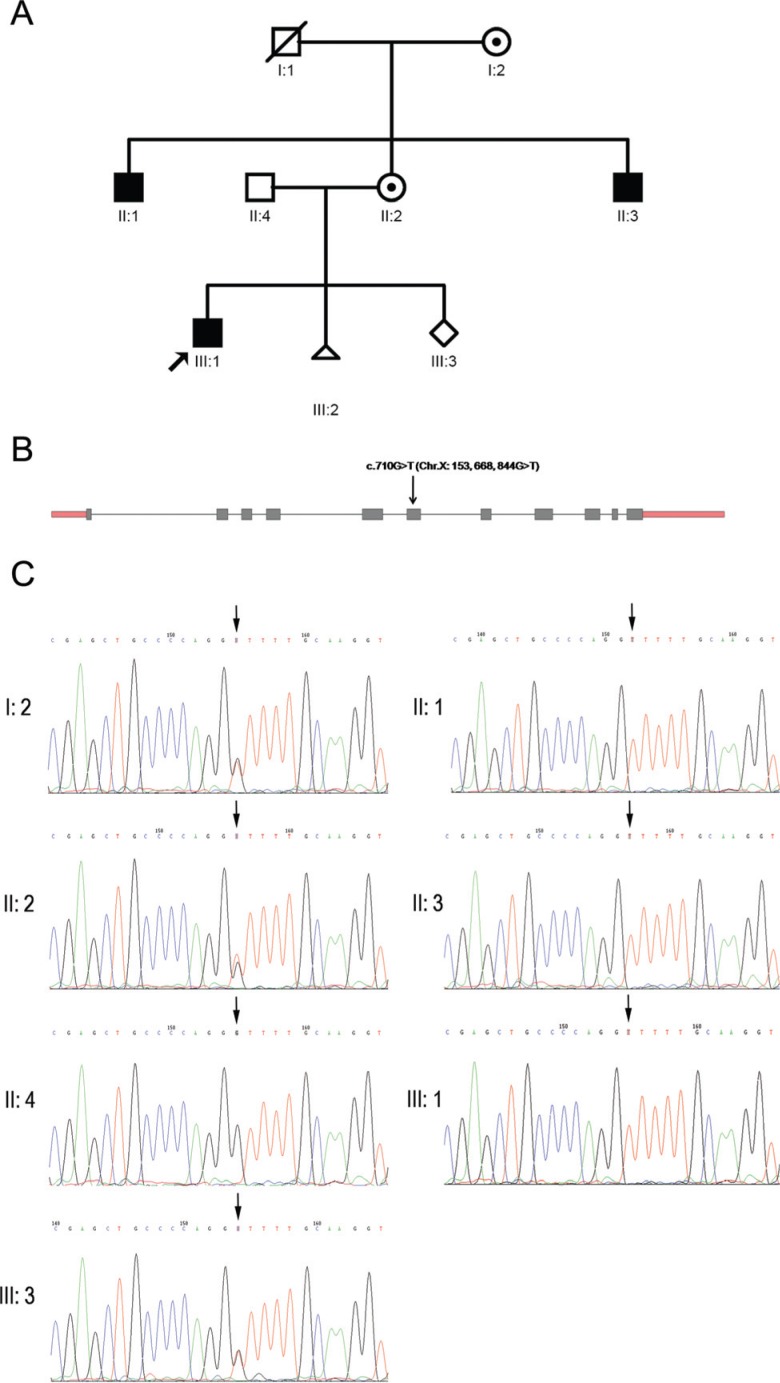
Pedigree and mutation. (A) Pedigree of the SZMRX Family. (B) Schematic of *GDI1* gene; arrow shows the c.710G > T mutation located at exon 6 of the *GDI1* gene (chr X: 153,668,844). **C.** The c.710G > T mutation segregating with the phenotype of non-syndromic X-linked intellectual disability (NS-XLID) in all male patients (II:1, II:3 and III:1) was validated by Sanger sequencing; arrow shows the locationof c.710 (chr X: 153,668,844).

This family, coded SZMRX, is of Chinese Han origin, with three generations including six members and one fetus (Figure 1A). There are three affected males belonging to two generations. The proband (III:1) is a 5-year old male, who was initially noted to have language developmental delay at 3 years old. His spoken language was limited to single words, like “father” and “mother”, and his emotions were expressed with purposeful body movements in most times. He was born after normal pregnancy and spontaneous delivery course, and his growth parameters were within the normal ranges of the Chinese reference for children's growth. He started to walk at the age of 18 months and his muscle tonus was within normal. Clinical, physical and mental examinations showed only a moderate intellectual disability, with verbal intelligence quotient (VIQ) = 44, performance IQ (PIQ) = 55, and full scale IQ = 45, evaluated by the Wechsler Preschool and Primary Scale of Intelligence III (WPPSI-III). The two maternal uncles of the proband (II:1 and II:3) presented the same phenotype: retardations were apparent in the first three years of life and the development of intelligence was non-progressive; there was no typical clinical manifestation other than the limitations in intellectual function and adaptive behaviors. The cranial magnetic resonance imaging (MRI) examination was performed in the 3 patients of this family and no abnormal anatomical feature was detected. Chromosomal aberrations and fragile X-syndrome were ruled out by G-banding karyotype examination and FMR1 mutation analysis (data not shown).

All obligate and possible carriers were of normal intelligence, and the pregnancy and delivery courses were uneventful in all female members. The mother of the proband got pregnant two years after his birth, but she terminated her pregnancy at the 12^th^ week for personal reasons. She was pregnant recently and the amniocentesis was executed at the 18^th^ week of this pregnancy. The pathological phenotype is considered inherited in X-linked recessive mode for several reasons. First, multiple affected members exist in continuous two generations reveal that the ID phenotype of the proband is not from a *de novo* mutation. Second, the normal phenotype of the proband's parents demonstrates that the ID phenotype is not inherited in an autosomal-dominant or X-linked dominant mode. Third, in consideration of the relatively rare allele frequency of ID-causing mutations, autosomal-recessive causes would be rather unlikely, as several individuals from two generations of this family with no consanguineous marriage were affected.

### DNA isolation

Genomic deoxyribonucleic acid (gDNA) was isolated from peripheral blood of the proband and all family members, and from the amniotic fluid of the fetus using the QIAamp DNA blood mini kit (Qiagen, Hilden, Germany).

### Whole exome sequencing

The proband underwent WES investigation, which was carried out on an Ion Torrent PGM platform (Life Technologies, Carlsbad, CA, USA). In brief, gDNA of the proband was fragmented by Ion Shear Plus Reagents to generate the fragment library. Exome capture was conducted by hybridizing the fragment library with biotin-labeled blocker at 47 °C for 72 h, followed by extraction using streptavidin-coated magnetic beads. Then the exome-enriched library was amplified by Ion TargetSeq Amplification Primer and purified using Agencourt AMPure XP Reagent (reagents mentioned above were all from Ion Plus Fragment Library Kit, Life Technologies). Thereafter, the template was produced by emulsion PCR (Ion PGM Template OT2 200 Kit) using the Ion OneTouch System (Life Technologies), and the sequencing program was executed on Ion 318 Chip V2 using the Ion PGM Sequencer (Life Technologies).

### Data analysis and validation

The whole exome sequencing data obtained from the Ion Torrent PGM platform were qualified as 98.76% of the exonic bases covered by at least 1 read, and 87.45% of those covered by 10 reads or more. The sequenced reads were aligned using the human reference genome (GRCh37/ hg19) as the reference sequence, then the aligned reads were applied to call variants by TVC4.2 and annotated by Ion Reporter 4.4 (Life Technologies). The alternative allele frequency (AAF) of all the variants was further adjusted by self-written script to browse the 1000 Genomes Project database, and common variants were excluded by filtering out those AAF > 0.01, in other words, only rare mutations were left for further investigation. Next, a script was executed to reserve only dangerous mutations, which were defined as variants that probably disrupt protein functions, such as frame shift indels, splice site variations, stop gain or loss, and deleterious missense(SIFT score < 0.05 or PolyPhen score > 0.5) (De Rubeis *et al.*, 2014). Finally, all the *in silico* predicted dangerous mutations were validated by Sanger sequencing in the proband, all family members and in the fetus as follows. The locations of interest in genomic DNA were amplified by PCR using primers designed by Primer Premier 5.0 (PREMIER Biosoft), shown in Supplementary Table S1. Sanger sequencing was carried out using the ABI Prism Big Dye Terminator Cycle Sequencing v3.1 Kit (Applied Biosystems, Foster City, CA, USA) on an ABI-3130xl genetic analyzer (Applied Biosystems). Co-segregation analysis was performed and mutations of X-linked recessive mode inheritance were considered because of the multiple affected members and the normal phenotype of the proband's parents.

### *In silico* analysis of the impact of amino acid conversion on the structure and molecular dynamics of GDI protein

The amino acid homology sequence alignment was performed using ClustalX to verify the evolutionary conservation of amino acid sequences we concerned between several vertebrates and human (Larkin *et al.*, 2007). The structure modeling work of both wild type and mutated protein was done by Modeller 9.11 on the basis of bovine GDP-Dissociation Inhibitor α-isoform (PDB ID: 1D5T), and assessed by SAVES. The structural effects of interested amino acid conversion were predicted by HOPE (Have (y)Our Protein Explained) website (Venselaar *et al.*, 2010). Molecular dynamics simulations of both wild type and mutated protein were conducted using a version of the PMEMD module from AMBER 12 (Case *et al.*, 2005).

## Results

Sixteen candidate variants (15 homozygous and 1 heterozygous, located in 12 genes) were identified from the proband by analysis of the WES data (Table 1). Subsequent Sanger validation followed by co-segregation analysis detected a c.710G > T (Chr.X: 153, 668, 844G > T) missense mutation, which mapped to exon 6 of the *GDI1* gene in all male patients (II: 1, II: 3 and III: 1), segregating with the phenotype of NS-XLID (Figure 1B, C). Meanwhile, the proband's mother and grandmother, who have a normal intellectual phenotype and social adjustment ability, were proved to be obligate carriers of this mutation (Figure 1C). Additionally, we also checked this variant by comparing to ExAC and 5000 Exomes (for URLs see the Internet Resources), and insured that they were not present in either of these two large scale exome consortia.

**Table 1:** Candidate mutations of the SZMRX family identified by whole exome sequencing#.

Gene	Genomic Positions (hg19)	Variant Type	Genotype	Nucleotide changes	Protein changes	Status
GALE	chr1:24,123,434	SNV	homozygous	Splice site 5.T>G		Not co-segregating
IDUA|SLC26A1	chr4:983,625	SNV	homozygous	c.1102 G>A	p. Gly368Ser	Not co-segregating
NDST1	chr5:149,907,466	SNV	homozygous	c.614 C>T	p. Pro205Leu	Not co-segregating
SLC17A5	chr6:74,331,619	SNV	homozygous	c.886 G>A	p. Val296Ile	Not co-segregating
AHI1	chr6:135,611,614	SNV	homozygous	c.3535 G>T	p. Asp1179Tyr	Not co-segregating
TG	chr8:133,931,735	SNV	homozygous	c.4493 C>T	p. Thr1498Met	Not co-segregating
DOCK8	chr9:312,134	SNV	homozygous	c.709G>A	p. Glu237Lys	Not co-segregating
FANCC	chr9:97,887,391	SNV	homozygous	c.973G>A	p. Ala325Thr	Not co-segregating
ABCC8	chr11:17,483,176	INDEL	homozygous	c.775_775delG	p. Ala259frame shift	Not co-segregating
SLC35C1	chr11:45,832,441	SNV	homozygous	c.611C>T	p. Thr204Met	Not co-segregating
TMEM216	chr11:61,165,741	SNV	heterozygous	c.440G>C	p. Arg147Thr	Not co-segregating
KMT2D	chr12:49,434,409	SNV	homozygous	c.7144C>T	p. Pro2382Ser	Not co-segregating
TSC2	chr16:2,133,765	SNV	homozygous	c.3953A>G	p. Glu1318Gly	Not co-segregating
CTSA|PLTP	chr20:44,526,704	SNV	homozygous	c.1369G>A	p. Gly457Ser	Not co-segregating
COL18A1|MIR6815	chr21:46,898,266	SNV	homozygous	c.1787C>T	p. Pro596Leu	Not co-segregating
GDI1	chrX :153,668,844	SNV		c.710G>T	p. Gly237Val	Co-segregating (X-linked recessive)

# Sixteen candidate mutations (located in 12 genes) were identified from the proband by analysis of the whole exome sequencing data, and finally a c.710 G > T (chrX: 153,668,844G > T) missense mutation, which mapped to exon 6 of the GDI1 gene (OMIM* 300104), was confirmed to be a pathogenic variant bySanger sequencing and co-segregation analysis in all male patients (II:1, II:3 and III:1).

**Figure 2:**
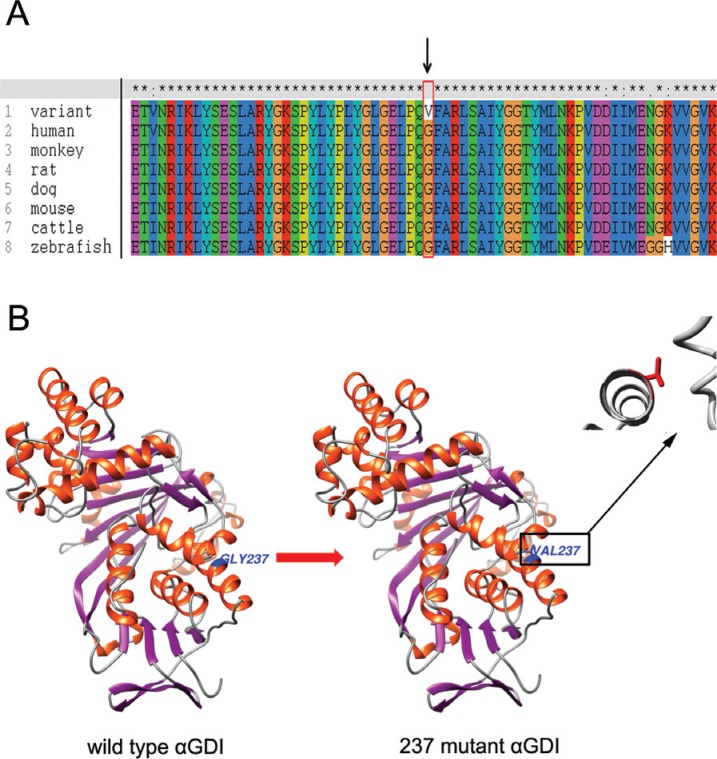
The GDI protein. (A)The evolutionary conservation of the respective amino acid sequences between several vertebrates and human, arrow points to the 237 residue.(B)The 237 position amino acid of GDI protein was changed from glycine to valine (p. Gly237Val).

**Figure 3:**
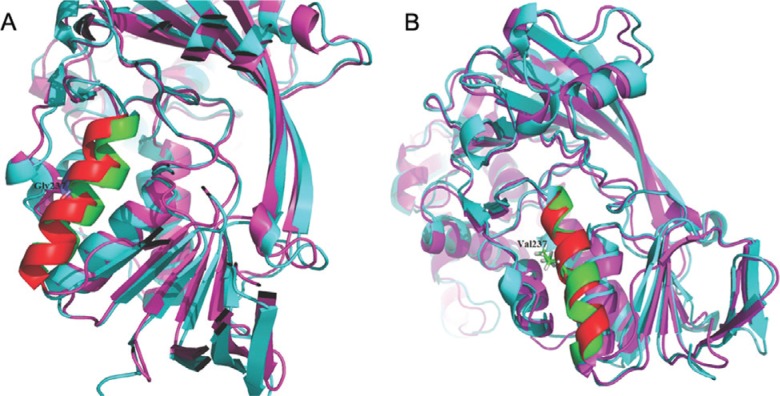
The 237 mutation. (A) The 237 residue located helix of the wild type αGDI exhibited a distinct conformational change after a 50 ns molecular dynamics simulation. (B) The conformation of this helix of 237 mutant αGDI showed relative stability *vs.* the wild type. The red color represents the initial conformation of 237 residue located helix of both wild type and 237 mutant αGDI, and green indicates the ones simulated by molecular dynamics.

This missense mutation changes the 237^th^position amino acid in the alpha-isoform GDP-Dissociation Inhibitor protein from glycine to valine (p. Gly237Val). Amino acid sequence alignment by ClustalX showed that the region, which p. Gly237Val mutant residue was situated in, was conserved across various vertebrates. Namely, neither this mutant residue (Val) nor any other residue with similar properties was observed at this position in other homologous sequences of vertebrates (Figure 2A). Several pathogenicity prediction score programs gave deleterious predictions for this p. Gly237Val mutation (Table S2). The computer built model of the GDI protein revealed that the location of residue 237 belongs to a hydrophobic domain composed by four helices, and substitution of glycine with valine introduces a larger side-chain into this four-helix hydrophobic pocket (Figure 2B). Molecular dynamics simulations lasting 50 nanoseconds (ns) also revealed that the residue 237 located in the helix of wild type αGDI exhibited a distinct conformational change after the minimization, heating and equilibration procedures (Figure 3A), while the p. Gly237Val mutant showed a relatively less obvious conformational change (Figure 3B).

## Discussion

Here we report a family (SZMRX) with three patients suffering from intellectual disability. Ultimately, the inheritance pattern of ID in this family was identified to be an X-linked recessive type by the WES and subsequent Sanger validation followed by co-segregation analysis, as the two unaffected females (mother-daughter relationship) share one rare heterozygous mutation related to XLID, and three consanguineous male patients are homozygous of the same loci.

The unique phenotype presented in the three patients is a moderate ID without any other recognizable clinical signs, and therefore the potential cause of this phenotype cannot be determined by G-banding karyotype examination and FMR1 mutation analysis. Whole exome sequencing was performed for the proband, and the probable pathogenic variants were validated by Sanger sequencing for all the family members and the fetus. Analysis of the WES data and subsequent Sanger validation followed by co-segregation analysis showed a missense mutation that segregated with the ID phenotype. This variant is a G to T transversion at position 710 of the coding sequence (c.710G > T), which is mapped to exon 6 of the *GDI1* gene, and it results in a p. Gly237Val substitution in the encoded protein. The *GDI1* gene contains 11 exons, spans 6.29 kb, and is located at chr. Xq28. This gene encodes the protein GDI, which belongs to the TCD/MRS6 family of GDP dissociation inhibitors, and serves as a regulator in the membrane-traffic process of vesicles by retrieving Rab (a protein family of Ras-like GTPases) from target membranes after a vesicular transport event (Pereira-Leal and Seabra, 2001). In brief, GDI has the capacity to bind the guanosine diphosphate (GDP)-bound form of Rab and to facilitate its release from the target membrane through forming cytosolic complexes. GDI subsequently delivers Rab to the cytoplasmic donor membrane, and after the disassembly of Rab-GDI complexes, GDI returns to the cytosol and becomes available for a new cycle (Pfeffer, 2013). In eukaryotes, exocytic and/or endocytic processes mediated by vesicles are major transport pathways between individual cells (Aridor and Balch, 1996). Rab proteins function as both directors, which point the specific transit way, and motors for vesicles to move between the membranes of cytoplasm and subcelluar organelles (Pfeffer, 2001; Goud, 2002). Meanwhile, the recycling of Rabs between the donor membrane and the target membrane needs cytosolic elements that act as guides for this traffic. Among Rab-interacting proteins, GDI not only serves as a retriever of Rabs, but also helps to maintain a pool of GDP-Rab in the cytoplasm (D'Adamo *et al.*, 2014). Thus, GDI has been considered a significant regulator for vesicular trafficking.

The phenotypes of ID caused by mutations of *GDI1* gene were first located in two ID families in 1998 (D'Adamo *et al.*, 1998). A Pro to Leu substitution was identified at the 92^nd^ position of the GDI protein's amino acid sequence in the MRX 41 family (OMIM#300849). This mutation affects a conserved residue in the α-helix beneath the Rab-binding platform of GDI protein (Schalk *et al.*, 1996) and subsequently decreases GDI affinity for Rab3A (a locally abundant Rab in brain) remarkably (Alory and Balch, 2001). A sequence-conserved region (SCR) named SCR3B (residues 232 to 259) was described as a vital part of the Rab-binding platform by studies on the X-ray structure of bovine brain GDI α-isoform (Schalk *et al.*, 1996). In that research, Glu233 and Arg240, which are near the 237th position mutant residue, were found to be critical for αGDI's binding capacity to Rab. Our genetic screening based on WES technique and subsequent *in silico* analysis located a pathogenic transition p. Gly237Val on an α-helix of human αGDI, which is buried in a hydrophobic pocket of the protein, just within this major functional SCR (residues 232 to 259) of GDI. Wildtype of the 237 residue is a glycine, which is the most flexible one among all amino acids. Meanwhile, the substitution of glycine with valine, which introduces a side-chain into this packed hydrophobic pocket between helices, results in numerous clashes with surrounding side-chains. Therefore, we speculate that the flexibility provided by glycine might be necessary for the protein's function, and the mutant residue (bigger and more hydrophobic than the wildtype) might disturb the binding capacity of GDI with Rabs. To verify our hypotheses, we carried out molecular dynamics simulations to exhibit the effect of the 237 residue variants on the function of αGDI. Our findings that the 237 position mutant αGDI exhibited a relatively less obvious conformational change than the wild type, confirm the loss of flexibility resulting from the substitution of glycine with valine. Hence, the Rab-binding capacity of GDI would probably be reduced by this restriction. Recently, new experimental evidence achieved by studies focusing on the αGDI mutated astrocytes revealed that impaired αGDI function could also affect the endolysosomal trafficking process in astrocytes. The mobility of vesicles in wild-type mouse astrocytes, which were transfected with two reported XLID-related αGDI mutations, was reduced (Potokar, *et al.*, 2016). Functional researches focusing on the detailed functional mechanism of this p. Gly237Val mutation are needed in future works.

In summary, our genetic analysis based on whole exome sequencing followed by Sanger validation revealed a novel missense mutation located in the *GDI1* gene in a Chinese family with three males suffering from XLID. Our findings extended the spectrum of *GDI1* mutations that cause X-linked non-specific intellectual disability (NS-XLID). Moreover, identification of the genetic cause for this NS-XLID family allowed us to assess the risk of the proband's mother for having an impaired child by prenatal testing during her pregnancy and to arrange appropriate management. Although the detailed functional mechanism and the frequency of this novel *GDI1* mutation await future investigations, we believe that the progressive identification of genetic defects associated with ID will eventually shed light on the underlying pathological mechanisms and help develop more effective treatment strategies for ID patients in the future.

## Supplementary material

The following online material is available for this article:

Table S1 - Characteristics of primers for amplification of candidate pathogenic regions on the genomic DNA

Table S2 - Possible consequence of *GDI1* p. Gly237Val mutation predicted by various algorithms
