# Supplementary material for: Exome sequencing identifies a novel mutation of the GDI1 gene in a Chinese non-syndromic X-linked intellectual disability family
Source: Genet Mol Biol. 2017 Aug 31;40(3):591–6. doi: 10.1590/1678-4685-GMB-2016-0249 (PMC5596370; doi:10.1590/1678-4685-GMB-2016-0249)
Supplement: Supplementary file 1 [file 1415-4757-gmb-1678-4685-GMB-2016-0249-Suppl01.pdf]

Supplementary Material to “Exome sequencing identifies a novel mutation of the GDI1 gene in a Chinese non-syndromic X-linked intellectual disability family"

Table S1.Characteristics of primers for amplification of candidate pathogenic regions on the genomic DNA.

Primer ID	Sequence of Primer F	Sequence of Primer R	Amplified regions	Size of PCR products
MRP-1	GATGTAATCCCGGACACCTG	AAGGGAAGGGACAGAGGAG	chr1:24123256-24123514	259 bp
MRP-3	TCCTGCCCTCCATTGACTG	AGCAGCACTGGCTCATAGGT	chr5:149907344-149907571	228 bp
MRP-5	CCTTGCTGACCATGCTTTC	CCACGGCCCTAGATCAAAG	chr6:135611487-135611749	263 bp
MRP-8	GGGGCACTCATTAGGAACC	GCCGGATTACTTGTTAAACGT	chr9:97887230-97887515	286 bp
MRP-9	TTGACCTAATGCCCTTTGAG	AAGGTAGGGAGTGGTGAGAAG	chr11:17483046-17483432	387 bp
MRP-10	CGTCCTCATCCCTCTTCCT	ATCTGCAGTCCTGTCACGTAG	chr11:45832275-45832654	380 bp
MRP-11	GGATTGCCTTTCCTGTTTAG	TGCTTGCCCAAATGTCTGT	chr11:61165578-61165894	317 bp
MRP-13	GGATGGCAGCAGTAAGCAG	CCCAAGTTCAGAGCCAGTTC	chr16:2133553-2133925	373 bp

Primer ID	Sequence of Primer F	Sequence of Primer R	Amplified regions	Size of PCR products
MRP-14	GGTAGCTGTGAGCAAGGATG	TGTTCGTGAGCTCCTGACC	chr20:44526587-44526994	408 bp
MRP-15	TGCATTTCCATGTAGACACTGT	GTGGCCCAAATGTCTCAAC	chr21:46898164-46898479	316 bp
MRP-16	GGAAGACTGGCCTGGTACCT	ACCCAAGGATGGAGTGAAGT	chrX:153668665-153668993	329 bp
