# Supplementary material for: Exome sequencing identifies a novel mutation of the GDI1 gene in a Chinese non-syndromic X-linked intellectual disability family
Source: Genet Mol Biol. 2017 Aug 31;40(3):591–6. doi: 10.1590/1678-4685-GMB-2016-0249 (PMC5596370; doi:10.1590/1678-4685-GMB-2016-0249)
Supplement: Supplementary file 2 [file 1415-4757-gmb-1678-4685-GMB-2016-0249-Suppl02.pdf]

Genetics and Molecular Biology

Supplementary Material to “Exome sequencing identifies a novel mutation of the GDI1 gene in a Chinese non-syndromic X-linked intellectual disability family"

Table S2. Possible consequence of GDI1 p.Gly237Val mutation predicted by various algorithms.

Algorithm	Score	Prediction
SIFT	0	deleterious
PolyPhen-2	1	probably damaging
Grantham	109	deleterious
PhyloP	2.39	conserved

Annotation:

A SIFT score predicts whether an amino acid substitution affects protein function, ranging from 0 to 1. Variants with scores closer to 0 are more confidently predicted to be deleterious, while scores very close to 1 are more confidently predicted to be tolerated. 0 to 0.05: deleterious; 0.05~1: tolerated (benign).

PolyPhen-2 score represents the probability that a substitution is damaging, ranging from 0 to 1 as well as SIFT, but with opposite meanings. 0 to 0.15: benign; 0.15 to 0.85: possibly damaging; 0.85 to 1: probably damaging.

Grantham score predicts the distance between two amino acids in an evolutionary sense, ranging from 5 to 215. A lower Grantham score reflects less evolutionary distance, a higher Grantham score reflects a greater evolutionary distance. Higher Grantham scores are considered

more deleterious.

PhyloP scores measure evolutionary conservation at individual alignment sites, ranging from -14 to 3. A positive score indicates slower evolution than expected, at sites that are predicted to be conserved. A negative score indicates faster evolution than expected, at sites that are predicted to be fast-evolving.
